# Impact of Interhospital Transfer on Outcomes in Acute Pancreatitis: Implications for Healthcare Quality

**DOI:** 10.3390/jcm13226817

**Published:** 2024-11-13

**Authors:** Tamara F. Kahan, Matthew Antony Manoj, Ankit Chhoda, Anabel Liyen Cartelle, Kelsey Anderson, Shaharyar A. Zuberi, Steven D. Freedman, Sunil G. Sheth

**Affiliations:** 1 Department of Medicine, Beth Israel Deaconess Medical Center, Boston, MA 02215, USA; tkahan@bidmc.harvard.edu (T.F.K.); aliyenca@bidmc.harvard.edu (A.L.C.); kander17@bidmc.harvard.edu (K.A.); shaharyar.zuberi@nyulangone.org (S.A.Z.); 2 Division of Gastroenterology, Department of Medicine, Beth Israel Deaconess Medical Center, Harvard Medical School, 330 Brookline Ave., Boston, MA 02215, USA; mmanoj1@bidmc.harvard.edu (M.A.M.); ankitchhoda@gmail.com (A.C.); sfreedma@bidmc.harvard.edu (S.D.F.)

**Keywords:** acute pancreatitis, quality of care, tertiary care center, community hospital

## Abstract

**Background/Objectives**: Effective management of acute pancreatitis (AP) hinges on prompt volume resuscitation and is adversely affected by delays in diagnosis. Given diverse clinical settings (tertiary care vs. community hospitals), further investigation is needed to understand the impact of the initial setting to which patients presented on clinical outcomes and quality of care. This study aimed to compare outcomes and quality indicators between AP patients who first presented to the emergency department (ED) of a tertiary care center and AP patients transferred from community hospitals. **Methods**: This study included AP patients managed at our tertiary care hospital between 2008 and 2018. We compared demographics and outcomes, including length of stay (LOS), intensive care unit (ICU) admission, rates of local and systemic complications, re-admission rates, and one-year mortality in transferred patients and those admitted from the ED. Quality indicators of interest included duration of volume resuscitation, time until advancement to enteral feeding, pain requiring opioid medication [measured in morphine milliequivalent (MME) dosing], and surgical referrals for cholecystectomy. Categorical variables were analyzed by chi-square or Fisher’s exact test; continuous variables were compared using Kruskal–Wallis tests. Regression was performed to assess the impact of transfer status on our outcomes of interest. **Results**: Our cohort of 882 AP patients comprised 648 patients admitted from the ED and 234 patients transferred from a community hospital. Transferred patients were older (54.6 vs. 51.0 years old, *p* < 0.01) and had less frequent alcohol use (28% vs. 39%, *p* < 0.01). Transferred patients had a significantly greater frequency of gallstone AP (40% vs. 23%), but a lower frequency of alcohol AP (16% vs. 22%) and idiopathic AP (29% vs. 41%) (*p* < 0.001). Regarding clinical outcomes, transferred patients had significantly higher rates of severe AP (revised Atlanta classification) (10% vs. 2% severe, *p* < 0.001) and ICU admission (8% vs. 2%, *p* < 0.001) and longer median LOS (5 vs. 4 days, *p* < 0.001). Regarding quality indicators, there was no significant difference in the number of days of intravenous fluid administration, or days until advancement to enteral feeding, pain requiring opioid pain medication, or rates of surgical referral for cholecystectomy. **Conclusions**: Though the quality of care was similar in both groups, transferred patients had more severe AP with higher rates of systemic complications and ICU admissions and longer LOS, with no difference in quality indicators between groups.

## 1. Introduction

Acute pancreatitis (AP) is a severe inflammatory disorder of the pancreas accounting for over 300,000 hospitalizations in the United States each year [1]. The financial burden of AP is substantial, with costs reaching up to $2.6 billion annually [2]. Despite a decline in mortality rates over the past decade, AP continues to present considerable mortality risks, particularly in high-risk groups; approximately 25% of patients develop severe AP which has a mortality rate of 20% [3].

As the incidence of AP has increased, recently reported to be at a rate of 2–5% per year, guidelines for early evidence-based interventions for management have been developed to mitigate AP-related mortality [4]. While AP has numerous etiologies that are managed differently, there are consistencies in the management of AP that are recommended regardless of etiology, namely intravenous fluid resuscitation and the prevention of local and systemic complications. Current ACG guidelines based on expert opinion and clinical trials recommend moderately aggressive fluid resuscitation with lactated ringer within the first 24 h of admission [5,6,7]. In patients with mild AP, oral feeding should be initiated based on patient hunger. The initiation of oral feeding within 24–48 h of presentation is associated with superior outcomes. Randomized controlled trials have demonstrated that there is no indication for the slow advancement of diet from clear liquids to solids [8].

Patients with AP present to community hospitals and tertiary care centers, with community hospitals vastly outnumbering tertiary care centers. Patients who initially present to a community hospital may be transferred to a tertiary care center, often for subspecialty care or prolonged intensive care that cannot be offered at the community hospital. Studies comparing outcomes of transferred patients with AP to patients admitted from the emergency department (ED) are limited and results are difficult to interpret, as higher mortality among transferred patients may reflect their increased illness severity rather than the care delivered at the community hospital or risks associated with discontinuity of care during the transfer process [9,10,11]. While community hospitals may not have robust advanced endoscopy resources or a team of gastroenterologists with expertise in pancreaticobiliary disease, the key early interventions—fluid resuscitation and nutritional support—can be delivered both in community settings and tertiary care [5].

Studies assessing outcomes for transferred and non-transferred patients with non-pancreatitis acute conditions have yielded mixed results. A multi-center study of 885,392 general adult inpatients found that transferred patients spend more time in intensive care units and have higher inpatient mortality [12]. This trend was corroborated and expanded upon in an analysis using National Inpatient Sample data, which included 1,397,712 transferred patients and 31,692,211 patients admitted from the ED and demonstrated that transferred patients had more frequent in-hospital adverse events and more complex discharge disposition [13]. However, the above analyses were limited by not being disease-specific and thus included transfers that occurred for specialty services or procedural intervention. Furthermore, both studies utilized administrative data and were not able to characterize patients as completely as would have been possible through chart review. While inter-hospital transfer confers risks related to discontinuity of care, results must be interpreted with acknowledgement of the increased severity and complexity of illness in this patient population. Within a nationally representative sample of Medicare claims for acute coronary syndrome, congestive heart failure, arrhythmia, sepsis, and pneumonia, among other conditions, transferred patients had greater resource utilization and variable results for 3-day and 30-day mortality. Patients transferred with acute coronary syndrome, sepsis, stroke, or respiratory disease had lower odds of 3-day and 30-day mortality. Patients transferred with esophageal or gastrointestinal disease, congestive heart failure, or renal failure, among other conditions, had greater odds of 3-day and 30-day mortality [14]. Finally, a study of outcomes of transferred and non-transferred patients with acute non-ST elevation myocardial infarctions in British Columbia found similar 30-day and 1-year mortality rates among both groups of patients.

Our study aimed to compare baseline characteristics and clinical outcomes between patients with AP admitted from the ED of a tertiary care center and patients with AP transferred from community hospitals in the referral network. Specifically, we sought to examine length of stay, in-hospital adverse events, intensive care unit (ICU) admissions, and mortality. We also compared quality indicators of care for AP. We hypothesize that transferred patients may have worse outcomes given the relative lack of resources at community hospitals and the tendency to transfer patients who have clinically worsened over their hospital stay. We aim to elucidate disparities in AP outcomes with the ultimate goal of guiding interventions to reduce these disparities.

## 2. Methods

### 2.1. Study Design and Setting

We conducted a retrospective cohort study of patients diagnosed with AP and hospitalized at a tertiary care center in the Boston area between 1 January 2008 and 31 December 2018. This study was approved by our center’s Institutional Review Board (Protocol ID: 2016P000158). This study is reported in accordance with the Strengthening the Reporting of Observational Studies in Epidemiology (STROBE) reporting guideline (Supplement S1) [15].

### 2.2. Patient Population

Adult patients (aged ≥ 18 years) were identified through International Classification of Diseases, Ninth and Tenth Revision codes 577.1 and K85.9 [16]. Electronic health records were then manually reviewed to confirm the diagnosis of AP. The diagnosis of AP was based on revised Atlanta classification (two of the following during hospitalization: typical abdominal pain, elevation of serum lipase level three times upper limit of normal, or evidence of pancreatitis on cross-sectional imaging) [17]. Patients who carried a diagnosis of chronic pancreatitis or pancreaticobiliary malignancy were excluded.

### 2.3. Data Collection

We collected demographic data including age, sex, race, ethnicity, body mass index (BMI), alcohol use, tobacco smoking history, and etiology of AP. Clinical data included comorbidities such as pre-existing DM, kidney disease, pulmonary disease, and cardiovascular disease quantified through the age-adjusted Charlson Comorbidity Index (CCI) and pain at time of presentation [measured through the visual analog scale (VAS)] [18]. The severity of AP at presentation was graded based on the revised Atlanta classification [coded as mild AP (no local or systemic complications), moderately severe AP (transient organ failure, local complications, or exacerbation of co-morbid disease) and severe AP (characterized by persistent organ failure for more than 48 h) [19]. The patients’ BISAP score, which predicts in-hospital mortality in AP based on clinical and laboratory variables, was calculated on presentation [20]. Opioid use during the entire hospitalization was quantified and converted into morphine milliequivalent (MME) units.

### 2.4. Outcomes of Interest

Outcome variables included length of stay, ICU admission, local complications of AP (i.e., pancreatic pseudocysts, peri-pancreatic fluid collections, pancreatic necrosis, and splenic vein thrombosis), systemic complications of AP (i.e., renal failure, acute hypoxemic respiratory failure requiring intubation, and sepsis), extra-pancreatic complications (i.e., alcohol withdrawal, gastrointestinal bleeding, and delirium), re-admissions within thirty days of discharge from index hospitalization, and mortality within one year of index hospitalization. Finally, we assessed quality indicators of care for AP including duration of volume resuscitation, time until advancement to enteral feeding, opioid use during the entire hospitalization [quantified and converted into morphine milliequivalent (MME) units], and surgical referrals for cholecystectomy, with the aforementioned quality indicators chosen based on a review of ACG guidelines and the literature [5,21,22].

### 2.5. Statistical Analysis

Categorical data are presented as proportions while continuous variables are reported as medians with interquartile ranges. Statistical differences in categorical variables were assessed using chi-square tests or Fisher’s exact tests, while continuous variables were analyzed using Kruskal–Wallis tests. Outcome variables (length of stay, ICU admission, local complications, systemic complications, extra-pancreatic complications, readmissions, and one-year mortality) served as dependent variables and were analyzed using univariate linear and logistic regression to identify associations with transferred or non-transferred status. Multivariate regression, controlling for age, sex, BMI, CCI, and AP etiology was planned for variables with *p* < 0.1 on univariate regression. All statistical analysis was performed using STATA software (StataCorp LLC, Version 17.0, College Station, TX, USA). A *p*-value < 0.05 was considered statistically significant.

## 3. Results

We identified 882 patients who met our inclusion criteria for AP and who did not carry a diagnosis of chronic pancreatitis or pancreaticobiliary malignancy. The sample included 648 patients admitted from the ED (73.4%) and 234 patients transferred from community hospitals (26.5%).

### 3.1. Demographics and Clinical Features

Baseline demographics and patient characteristics of the study cohort are summarized in Table 1. The mean age of the full cohort was 51.8 years (SD 21.2); patients admitted from the ED were significantly younger than transferred patients (51.0 vs. 54.6, *p* < 0.01). The cohort was 49% female and 73% Caucasian; median BMI was 26.3 (7.1) kg/m^2^. There were no statistically significant differences in distribution of sex, race, or BMI between the two groups. We observed a substantial psychosocial burden within our study sample, with 310 patients (36%) reporting active alcohol use and 241 patients (27%) reporting active smoking at time of admission. Notably, active alcohol use was significantly more prevalent in patients admitted from the ED compared to transferred patients (39% vs. 28%, *p* < 0.01). The median CCI of patients was 1; there was no difference in CCI when comparing transferred patients to patients admitted from the ED.

### 3.2. Pancreatitis Etiology and Severity

The etiology of AP varied between groups, with transferred patients having significantly higher rates of gallstone pancreatitis (40% vs. 23%) and post-ERCP pancreatitis (4% vs. 2%), and lower rates of alcoholic pancreatitis (16% vs. 22%) and idiopathic pancreatitis (29% vs. 41%) compared to patients admitted from the ED (*p* < 0.01). On presentation, out of the full sample of 882 patients, 642 patients (76%) met criteria for mild pancreatitis, 172 patients (20%) met criteria for moderate pancreatitis, and 34 patients (4%) met criteria for severe pancreatitis by revised Atlanta classification. Transferred patients had significantly higher rates of moderate (32% vs. 16%) and severe (10% vs. 2%) pancreatitis (*p* < 0.01). Median BISAP score was 1 among both patients admitted from the ED and transferred patients.

### 3.3. Clinical Outcomes

#### 3.3.1. Length of Stay and Intensive Care Unit Admission

Transferred patients had a significantly longer median length of stay (5 vs. 4 days, *p* < 0.001) and were more likely to be transferred to the ICU (8% vs. 2%, *p* < 0.001) compared to patients admitted from the ED (Table 2). These associations were statistically significant on regression analysis (Table 3). When controlling for age, sex, BMI, CCI, and AP etiology, transferred patients had significantly longer lengths of stay (OR 3.09; 95%CI 2.08–4.10) and were significantly more likely to be transferred to the ICU compared to patients admitted from the ED (OR 7.05; 95%CI 3.22–15.44) (Table 4).

#### 3.3.2. Local and Systemic Complications

We compared the frequency of local complications of AP, including pancreatic pseudocysts, peri-pancreatic fluid collections, pancreatic necrosis, and splenic vein thrombosis, between transferred patients and patients admitted from the ED. There was no difference in the rates of any of these local complications between groups. We also assessed systemic complications of AP, including renal failure, acute hypoxemic respiratory failure requiring intubation, and sepsis. Transferred patients had significantly higher rates of systemic complications overall (29% vs. 7%, *p* < 0.001), and higher rates of each complication when analyzed individually (*p* < 0.001). On multivariate regression, transferred patients were significantly more likely to have systemic complications of AP (OR 4.22; 95% CI 2.61–6.84) (Table 4). Regarding extra-pancreatic complications, there was no difference between patients admitted from the ED and transferred patients when considering all extra-pancreatic complications (i.e., alcohol withdrawal, gastrointestinal bleeding, and delirium) in aggregate though when considered individually, patients admitted from the ED had higher rates of alcohol withdrawal (5.7% vs. 1%, *p* < 0.01) and lower rates of delirium (1.5% vs. 6%, *p* < 0.01) compared to transferred patients.

#### 3.3.3. Thirty-Day Re-Admissions and One-Year Mortality

Overall, 166 (19%) patients were re-admitted within 30-days of discharge; re-admission rates did not vary between transferred patients and patients admitted from the ED. One-year mortality in the sample was 2.5%; one-year mortality was not significantly different when comparing transferred patients and patients admitted from the ED.

### 3.4. Quality of Care Measures

An initial pain assessment score using VAS demonstrated a median score of seven (IQR: 4). Regarding pain management with opioids, patients received a median of 8.5 MME within the first 24 h of admission and 14 MME over the course of admission. Regarding IV fluid resuscitation, 554 patients (62.8%) of patients received IV fluids within four hours of presentation and patients received a median of 3 days (IQR: 2) of IV fluids. Diet was advanced to oral nutrition in a median of 2 days (IQR: 2). On discharge, 169 patients (19.1%) received a surgical referral for cholecystectomy. There was no significant difference in any of the above measures when comparing transferred patients to patients admitted from the ED.

## 4. Discussion

We found numerous differences in baseline characteristics and outcomes for patients with AP when comparing those transferred from community hospitals to those who presented to the ED of our tertiary care center. We found that transferred patients had a higher incidence of moderate and severe AP and higher rates of gallstone-related and post-ERCP pancreatitis. Patients who presented to the ED had a higher baseline rate of active alcohol use and higher rates of alcohol-related pancreatitis. Transferred patients had longer lengths of stay, higher rates of admission to the ICU, and higher rates of systemic complications of pancreatitis compared to patients admitted from the ED.

There are numerous possible explanations for the above findings. The higher incidence of moderate and severe pancreatitis, longer lengths of stay, higher rates of admission to the ICU, and higher rates of systemic complications of transferred patients may represent greater illness severity among transferred patients or suboptimal initial management at community hospitals; however, there were no differences in the quality of care between groups. Reassuringly, mortality rates were not significantly different between transferred patients and patients admitted from the ED, reflecting uniform high-quality care delivered at our tertiary care pancreas center. Unfortunately, we did not have access to data on fluid resuscitation delivered at community hospitals, with fluid resuscitation being one of the cornerstones of management in AP. Inadequate fluid resuscitation is associated with the development of necrotizing pancreatitis and consequent multi-organ failure [23,24]. Data support that delayed presentation for acute pancreatitis is associated with greater pancreatitis severity at the time of presentation, higher rates of organ failure, and greater likelihood of requiring procedural intervention [25].

Our study has a number of strengths. This study is strengthened by rigorous clinical follow-up, detailed clinical history, and meticulous work-up for each AP patient in a large sample of over 800 patients with AP. Manual chart review ensures a high degree of accuracy [26]. The retrospective design of the study mitigates the “Hawthorne effect”, providing a more accurate reflection of real-time practices. We have included a wide range of outcome variables including systemic and extra-pancreatic complications of AP, which are responsible for a substantial mortality and morbidity burden.

Despite these strengths, there are a number of limitations which should be noted. First, these findings stem from the population of a large single-center tertiary referral center, and thus may not be generalizable to all other hospital systems. Second, our current sample size limits the ability to detect small differences between groups. Third, our results may reflect referral bias, with transfer patients having more severe AP at the time of transfer. Last, as a retrospective study, although many clinical and demographic variables were captured, the likelihood of unmeasured confounding always exists.

In conclusion, we found that relative to patients admitted from the ED of a tertiary care center, transferred patients had longer lengths of stay, higher rates of admission to the ICU, and higher rates of systemic complications though readmissions within 30-days of discharge and one-year mortality was not significantly different between groups, indicating that high quality care delivered by our pancreas center serves to mitigate possible greater illness severity or suboptimal management of transferred patients prior to their transfer. Further prospective studies are needed to identify reasons for disparities in outcomes between the two groups of AP patients.

## Figures and Tables

**Table 1:** Demographic and clinical characteristics of patients admitted from the ED and transferred patients with acute pancreatitis.

Demographics				
	Total (N = 882)	Patients Admitted from ED (N = 648)	Transferred Patients (N = 234)	*p*-Value
Age [mean (SD)]	51.8 (21.2)	51.0 (20.6)	54.6 (21.8)	<0.01
Body Mass Index [M (IQR)]	26.3 (8.3)	26.0 (8.7)	26.7 (7.1)	0.54
Sex [Female n (%)]	431 (49%)	310 (48%)	121 (52%)	0.31
Race [Caucasian n (%)]	633 (73%)	460 (72%)	173 (78%)	0.1
Active Alcohol use [n (%)]	310 (36%)	246 (39%)	64 (28%)	<0.01
Active Tobacco Use [n (%)]	241 (27%)	187 (29%)	54 (23%)	0.08
Revised Atlanta Classification [mild/moderate/severe: n (%)]	642 (76%)	518 (82%)	124 (57%)	<0.001
	172 (20%)	102 (16%)	70 (32%)	
	34 (4%)	12 (2%)	22 (10%)	
Etiology of AP [n (%)]				<0.001
Gallstone	237 (27%)	145 (23%)	92 (40%)	
Alcohol	179 (20%)	143 (22%)	36 (16%)	
Post-ERCP	24 (3%)	14 (2%)	10 (4%)	
Other ^1^	102 (13%)	81 (12%)	28 (11.5%)	
Idiopathic	327 (37%)	261 (41%)	66 (29%)	
Charlson Comorbidity Index [M (IQR)]	1 (1)	0 (1)	1 (2)	0.3
BISAP [M (IQR)]	1 (0)	1 (0)	1 (1)	<0.001

^1^ Includes hypertriglyceridemia, medication-induced, autoimmune pancreatitis, pancreatic mass, and acute on chronic acute pancreatitis.

**Table 2:** Clinical outcomes and quality of care measures of patients admitted from the ED and transferred patients with acute pancreatitis.

**Clinical Outcomes**				
	**Total (N = 882)**	**Patients Admitted from ED (N = 648)**	**Transferred Patients (N = 234)**	***p*-Value**
Length of Stay (days) [M (IQR)]	4 (3.6)	4 (4)	5 (5.8)	<0.001
ICU transfer [n (%)]	29 (3%)	10 (2%)	19 (8%)	<0.001
Local complications (defined as one or more of the following: pseudocysts, peri-pancreatic fluid collections, pancreatic necrosis, and splenic vein thrombosis) [n (%)]	128 (15%)	89 (14%)	39 (17%)	0.28
Systemic complications (defined as one or more of the following: renal failure, acute hypoxemic respiratory failure requiring intubation, and sepsis) [n (%)]	112 (13%)	43 (7%)	69 (29%)	<0.001
Renal failure	52 (6%)	22 (3%)	30 (13%)	<0.001
Acute hypoxemic respiratory failure requiring intubation	18 (2%)	4 (0.6%)	14 (6%)	<0.001
Sepsis	41 (5%)	16 (2%)	25 (11%)	<0.001
Extra-pancreatic complications (including alcohol withdrawal, gastrointestinal bleeding, and delirium) [n (%)]	83 (9%)	61 (9%)	22 (9%)	0.99
Alcohol withdrawal [n (%)]	39 (4%)	37 (6%)	2 (1%)	<0.01
Delirium [n (%)]	24 (3%)	10 (2%)	14 (6%)	<0.01
Re-admitted within 30-days of discharge [n (%)]	166 (19%)	131 (20%)	35 (11%)	0.08
One-year mortality [n (%)]	22 (3%)	13 (3%)	9 (5%)	0.15
**Quality of Care Measures**				
	**Total (N = 882)**	**Patients Admitted from ED (N = 648)**	**Transferred Patients (N = 234)**	***p*-Value**
Days of IV fluid resuscitation [M (IQR)]	3 (2)	3 (2)	3 (3)	0.69
Initial pain assessment through visual analog scale (VAS) [M (IQR)]	7 (4)	7 (4)	7 (4)	0.38
MME within first 24 hours of admission [M (IQR)]	8.5 (14)	8.5 (16)	8.5 (10)	0.79
Total MME over admission [M (IQR)]	14 (44.6)	12 (41)	16 (59.8)	0.36
Time to PO nutrition (days) [M (IQR)]	2 (2)	2 (2)	2 (3)	0.44
Received IV fluids within 4 hours of admission [n (%)]	554 (81%)	413 (82%)	141 (78%)	0.32
Surgical referral for cholecystectomy [n (%)]	169 (19%)	104 (16%)	65 (28%)	0.57

**Table 3:** Univariate linear and logistic regression analysis assessing the association of transfer status with various outcomes of AP (n = 882).

Parameter	Odds Ratio	*p*-Value
Length of stay	3.09 (95%CI: 2.08–4.10)	<0.001
ICU admission	7.05 (95%CI: 3.22–15.44)	<0.001
Local complications	1.14 (95%CI: 0.74–1.75)	0.57
Systemic complications	4.22 (95%CI: 2.61–6.84)	<0.001
Extra-pancreatic complications	1.05 (95%CI: 0.61–1.80)	0.86
Re-admission within 30 days	0.86 (95%CI: 0.57–1.29)	0.46
One-year mortality	1.55 (95%CI: 0.47–5.11)	0.56

**Table 4:** Multivariate regression analysis assessing the association of transfer status with various outcomes of AP, controlling for age, gender, BMI, CCI, and etiology of AP (n = 882).

Parameter	Odds Ratio	*p*-Value
Length of stay	3.16 (95%CI: 1.99–4.33)	<0.001
ICU admission	8.23 (95%CI: 3.26–20.72)	<0.001
Systemic complications	3.75 (95%CI: 2.01–5.89)	<0.001

## Data Availability

The original contributions presented in the study are included in the article/Supplementary Materials, further inquiries can be directed to the corresponding author.

## Supplementary Materials

The following supporting information can be downloaded at: https://www.mdpi.com/article/10.3390/jcm13226817/s1, Supplement S1: Strengthening the Reporting of Observational Studies in Epidemiology (STROBE) Statement.
